# Supplementary material for: A high-resolution mRNA expression time course of embryonic development in zebrafish
Source: eLife. 2017 Nov 16;6:e30860. doi: 10.7554/eLife.30860 (PMC5690287; doi:10.7554/eLife.30860)
Supplement: Supplementary file 6. [file elife-30860-supp6.zip › biolayout-clusters-files/Cluster252-genes.html]

Cluster252


# Cluster252: Genes

| Ensembl ID | Gene Name | Chr | Start | End | Biotype |
| --- | --- | --- | --- | --- | --- |
| ENSDARG00000075327 | dock10 | 15 | 1933803 | 2079084 | protein\_coding |
| ENSDARG00000035650 | evla | 17 | 30883528 | 30959015 | protein\_coding |
| ENSDARG00000001710 | flot1a | 19 | 7019364 | 7038479 | protein\_coding |
| ENSDARG00000016676 | gnao1a | 18 | 22804399 | 22987598 | protein\_coding |
| ENSDARG00000043821 | klf7b | 9 | 28530363 | 28588325 | protein\_coding |
| ENSDARG00000059972 | raly | 6 | 50023497 | 50205029 | protein\_coding |
